# Supplementary material for: Patterns of Variation and Chemosystematic Significance of Phenolic Compounds in the Genus Cyclopia (Fabaceae, Podalyrieae)
Source: Molecules. 2019 Jun 26;24(13):2352. doi: 10.3390/molecules24132352 (PMC6651507; doi:10.3390/molecules24132352)
Supplement: Supplementary file 1 [file molecules-24-02352-s001.zip › Supplementary Figures.docx]

**
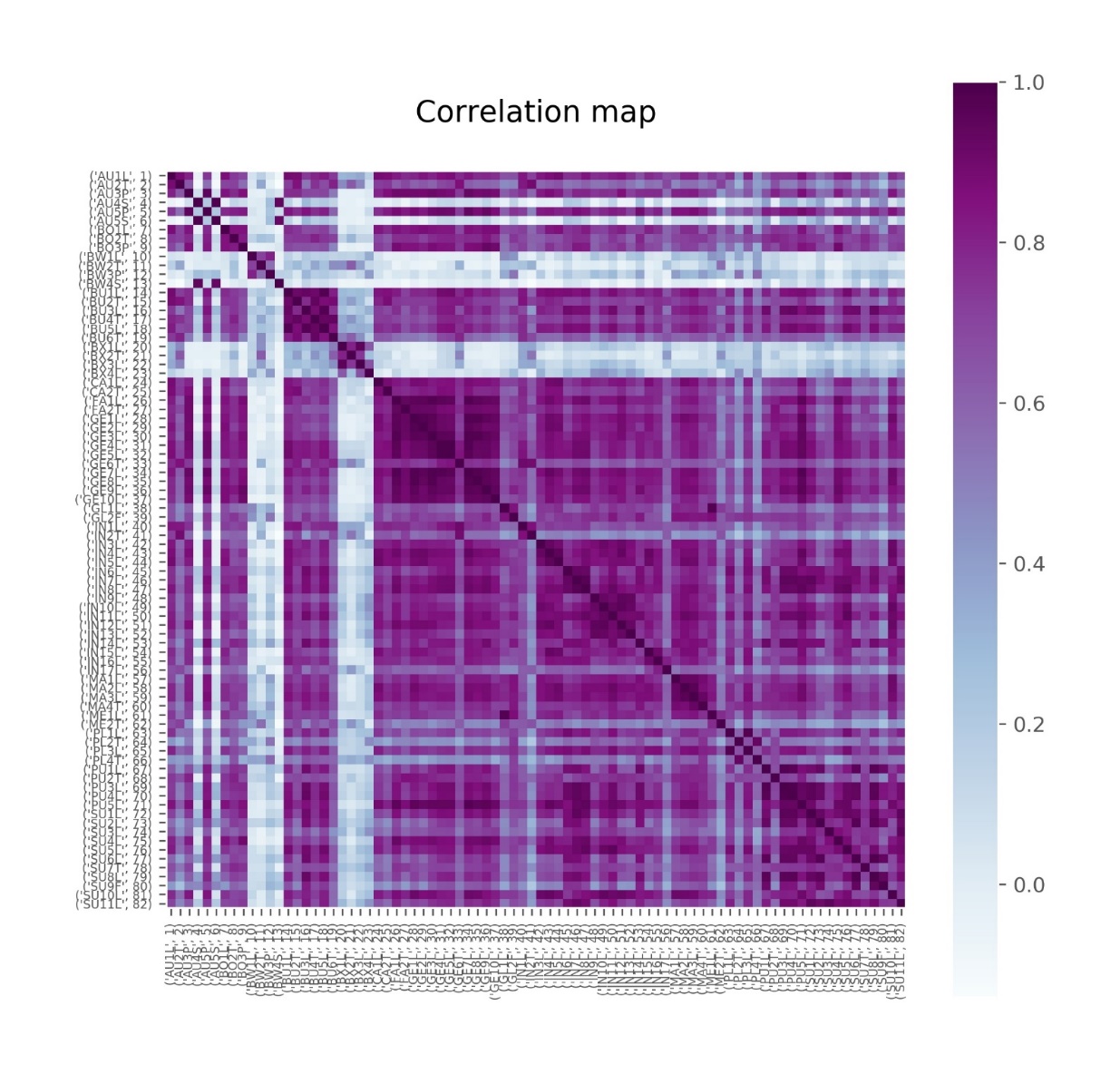
 Figure S1.** Correlation map between extracts of *Cyclopia* samples based on LCMS data
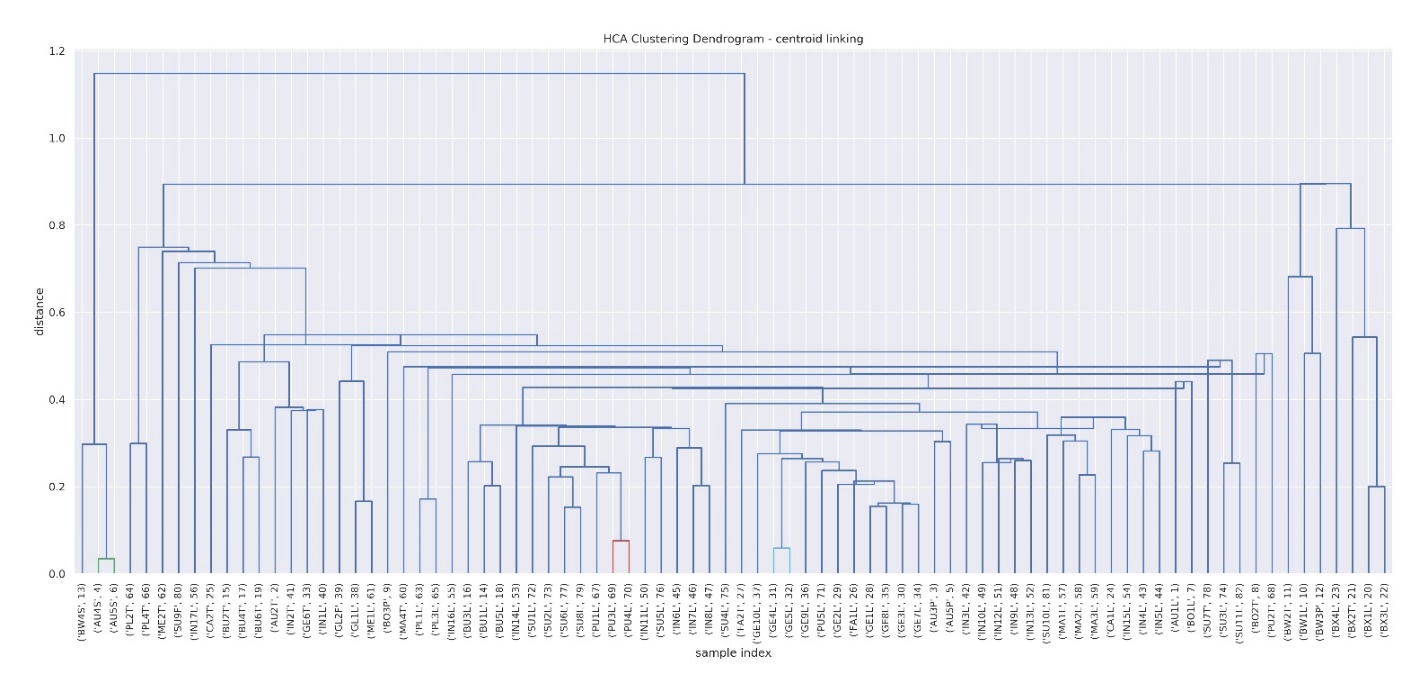
 **Figure S2**. Dendogram showing the relations of the different *Cyclopia* extracts based on Electrospray LCMS data.
